# Universal scaling rules predict evolutionary patterns of myogenesis in species with indeterminate growth

**DOI:** 10.1098/rspb.2011.2536

**Published:** 2012-01-11

**Authors:** Ian A. Johnston, Bjarni K. Kristjánsson, Charles G. P. Paxton, Vera L. A. Vieira, Daniel J. Macqueen, Michael A. Bell

**Affiliations:** 1 School of Biology, Scottish Oceans Institute, University of St Andrews, St Andrews, KY16 8LB, UK; 2 Department of Aquaculture and Fish Biology, Hólar University College, Háeyri 1, 550 Sauðárkrókur, Iceland; 3 Centre for Research into Ecological and Environmental Modelling, School of Mathematics and Statistics, University of St Andrews, St Andrews, Fife KY16 9LZ, UK; 4 Department of Ecology and Evolution, State University of New York, Stony Brook, NY 11794-5245, USA

**Keywords:** parallel evolution, dwarfism, muscle fibres, threespine stickleback, Arctic charr, scaling laws

## Abstract

Intraspecific phenotypic variation is ubiquitous and often associated with resource exploitation in emerging habitats. For example, reduced body size has evolved repeatedly in Arctic charr (*Salvelinus alpinus* L.) and threespine stickleback (*Gasterosteus aculeatus* L.) across post-glacial habitats of the Northern Hemisphere. Exploiting these models, we examined how body size and myogenesis evolve with respect to the ‘optimum fibre size hypothesis’, which predicts that selection acts to minimize energetic costs associated with ionic homeostasis by optimizing muscle fibre production during development. In eight dwarf Icelandic Arctic charr populations, the ultimate production of fast-twitch muscle fibres (FN_max_) was only 39.5 and 15.5 per cent of that in large-bodied natural and aquaculture populations, respectively. Consequently, average fibre diameter (FD) scaled with a mass exponent of 0.19, paralleling the relaxation of diffusional constraints associated with mass-specific metabolic rate scaling. Similar reductions in FN_max_ were observed for stickleback, including a small-bodied Alaskan population derived from a larger-bodied oceanic stock over a decadal timescale. The results suggest that in species showing indeterminate growth, body size evolution is accompanied by strong selection for fibre size optimization, theoretically allowing resources saved from ionic homeostasis to be allocated to other traits affecting fitness, including reproduction. Gene flow between small- and large-bodied populations residing in sympatry may counteract the evolution of this trait.

## 1. Introduction

Intraspecific phenotypic variation is an important aspect of observed biological diversity and is often associated with resource polymorphism, a phenomenon involving divergent use of habitat resources [1,2]. Numerous examples of resource polymorphism have been documented in fish, amphibians and birds, accounting for distinct morphs and/or species that may differ in behaviour, life-history traits and morphology (reviewed in Smith & Skúlason [2]). Resource polymorphism is an important factor in ecological speciation [3] and is particularly prevalent in post-glacial freshwater habitats of the Northern Hemisphere, where it probably underlies the striking intraspecific phenotypic diversity observed in several fish lineages, including salmonids, smelt and stickleback [2]. Natural selection is believed to be important in shaping the evolution of resource polymorphism, and consequently it is common for similar fish phenotypes to arise independently in geographically discrete habitats sharing similar ecological features [2]. Such parallel evolution has been commonly observed for a number of teleost traits including adult body size [2,4]. Understanding the evolution of finer scale traits associated with intraspecific diversity in body size is of interest because of the powerful constraints on structure and function imposed by universal scaling laws [5].

Myogenesis represents a trait that is closely associated with intraspecific diversity in body size in salmonid fish. In particular, the ultimate number of muscle fibres generated during development (FN_max_) is probably under divergent selection between dwarf and relatively larger populations of Arctic charr [6] and Atlantic salmon (*Salmo salar* L.) [7]. This phenomenon has been interpreted in terms of the energetic costs of maintaining ionic homeostasis in muscle cell membranes, which requires the optimization of muscle fibre size [6,7]. The optimum fibre size (OFS) hypothesis predicts that fibre number is adjusted in a trade-off between avoiding diffusional constraints and ionic homeostasis costs, which are theoretically proportional to the surface to volume ratio of individual fibres [6,7]. In wider support of the OFS hypothesis, the adaptive radiation of notothenioid perciform fish within the Antarctic Ocean was accompanied by a reduction in FN_max_ and the evolution of ‘giant muscle fibres’, probably reflecting a relaxation of diffusional constraints at low temperatures [8]. Further, in the American lobster (*Homarus americanus*), the density of sodium pumps, levels of Na^+^K^+^ ATPase activity and associated metabolic costs of ion pumping were twofold higher in small- than large-diameter muscle fibres, matching a doubling in surface to volume ratio [9]. This latter finding validates a central assumption of the OFS hypothesis in terms of the energetic costs of maintaining different-sized fibres. Despite these observations, the quantitative predictions of the OFS hypothesis and its ability to explain evolutionary patterns of myogenesis remain largely unexplored.

This study examines general principles surrounding the OFS hypothesis and the associated evolution of myogenesis and body size in Arctic charr and three-spine stickleback. In both lineages, oceanic populations have repeatedly invaded freshwater habitats arising since the end of the last Ice-Age and diversified into demes with distinctive morphological characteristics and a range of body sizes [10–12]. More than 35 dwarf populations of Arctic charr have been documented in Iceland, occurring in allopatry or sympatry with larger-bodied morphs in spring-fed rivers, lakes and ponds [13–16]. Despite substantial genetic differentiation between modern populations, there has been very restricted gene flow between waterways, indicating that habitats have typically remained isolated following the initial invasion [16]. Microsatellite studies suggest that the dwarf charr phenotype has arisen repeatedly by selection, with a relatively minor role for non-selective evolutionary processes [16]. A similar pattern of post-glacial radiation and parallel phenotypic evolution has been observed and extensively studied in stickleback residing in fresh water habitats around Cook Inlet, Alaska [10,11]. This includes a small-bodied population that was derived from a large-bodied sea-run population sometime between 1983 and 1988 [17,18]. By exploiting these two distantly related teleost models of intraspecific phenotypic diversification, we were able to investigate predictions of the OFS hypothesis within a broad ecological context that is widely applicable to species with indeterminate muscle growth.

## 2. Material and methods

Fish fork length (tip of snout to fork in tail) was measured in all cases and is hereafter referred to as length.

### (a) Arctic charr

**Figure 1.**
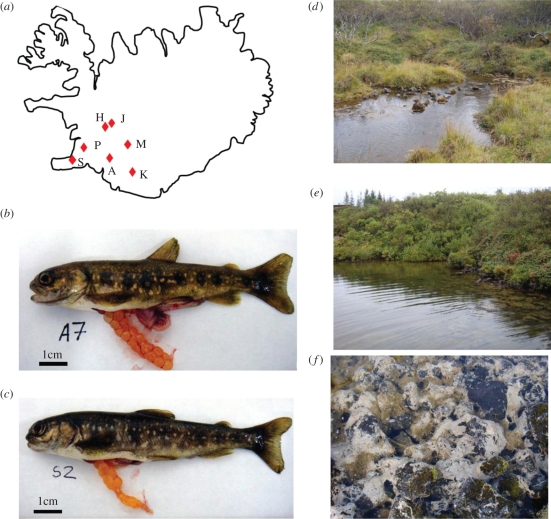
(a) Map of Iceland showing the locations of each of the Arctic charr populations studied: Álftavatn (A); Straumsvík (S); Hrauná (H), Sílatjörn (J), Kaldárbotnar (K), Silungapollur (P) and Miðhúsaskógur (M). (*b*) Dwarf charr from Álftavatn. (*c*) Dwarf charr from Straumsvík. (*d*) Hrauna, a spring-fed stream habitat, (*e*) Straumsvík, a spring-fed pond habitat and (*f*) Straumsvík substratum showing the network of spring-fed volcanic lava fissures.

Dwarf Arctic charr were caught using electrofishing at various locations around Iceland during August 2009 (figure 1*a*). Hrauná (H) (64°42′ N: 20°59′ W) is a spring-fed stream habitat, whereas Álftavatn (A) (64°01′ N: 20°57′ W), Kaldárbotnar (K) (64°41′ N: 20°51′ W), Miðhúsaskógur (M) (64°02′ N: 20°35′ W), Straumsvík (S) (64°02′ N: 22°02′ W), Silungapollur (P) (64°02′ N: 22°02′ W) and Sílatjörn (J) (64°42′ N: 20°58′ W) are spring-fed ponds. Around 200–250 individuals were electrofished from each population and the largest 50–80 fish retained. All habitats were characterized by a complex substrate of lava rock and average temperatures of 4–5°C (see Kristjánsson [15] for further information about the physical characteristics of each location). Fish were transported to Verið Holar University College research station and the largest individuals (nine per population, approximately representing the 95th percentile) were held in a recirculating aquarium at comparable temperatures to the wild (ranging from 3 to 6°C) and sampled within one week for histology. The remaining fish were passive inductive transponder-tagged (12 mm tags, Sokymat Automotive) after 10 days and maintained for 252 days in four 1 m^3^ tanks, each receiving the same water supply, heated to an elevated temperature compared with the wild (ranging from 6 to 12°C) and having the same photoperiod (12 L : 12 D) and feeding regimes (daily satiation feeding with bloodworms and a commercial diet [Laxa LF23]). In order to minimize cannibalism, individuals were spread across the four tanks according to their body size, such that each population was distributed between two to three tanks. The nine largest laboratory-reared dwarfs were sampled for histology in May 2010. The large-bodied aquaculture charr population (length, 45.3 ± 0.9 cm, body mass 1540 ± 60 g, mean ± s.e., *n* = 6) comprised a mixture of generalist Arctic charr derived from seven wild populations (further details available on request to I.A.J.). The founding fish were taken into aquaculture between 1989 and 1992 and subjected to six generations of selection for high body mass at age and late sexual maturation. All fish were killed using a Schedule 1 method in compliance with UK Home Office guidelines. The sex and maturation statuses of each fish were recorded.

Muscle fibre number and size were determined from a 4–5 mm steak at 0.7 length, which was photographed at high resolution. The right-hand side of this standard muscle cross section was divided into one to three numbered blocks. Blocks were frozen in 2-methyl-butane cooled to freezing with liquid nitrogen and 8 μm frozen sections cut at −20°C in a cryostat (CM1850, Leica Instruments GmbH, Heidelberg, Germany). Sections were stained with modified Harris haematoxylin solution (Sigma-Aldrich, Chemie GmbH, Steinberg, Germany) and myosin ATPase activity with and without alkaline (pH 10.4) pre-incubation in order to differentiate between slow and fast muscle fibres [6]. Measurements of fibre diameter (FD) were routinely conducted on haematoxylin-stained sections, which provided better definition of the fibre sarcolemma. Sections were examined with an Axioskop 2 microscope (Zeiss) and 8–10 fields/block photographed with a 10× objective using an AxioCam HRC and Axiovision software (Zeiss). The total cross-sectional area of fast muscle and the outlines of 800–1000 individual fast muscle fibres per fish were digitized using SigmaScan Pro software (Systat Software, Inc, Point Richmond, CA, USA). The total number of fast muscle fibres per trunk cross section was estimated as previously described [6].

### (b) Threespine stickleback

Fish were collected from lakes in the Matanuska-Susitna Borough of Cook Inlet, Alaska, USA in June 2007 using unbaited minnow traps. Large-bodied populations were sampled from Rabbit Slough (RS) (61°32′ N, 149°16′ W), Frog Lake (FL) (61°61′ N, 149°71′ W) and Seymour Lake (SL) (61°36 N, 149°39′ W). Small-bodied populations were sampled from Mud Lake (ML) (61°56″ N, 148°94′ W) and Loberg Lake (LL) (61°33 N, 149°15′ W). The RS population is anadromous [19] and the other populations are freshwater residents. LL represents a rapidly evolving population derived from a large-bodied sea-run population between 1983 and 1988 [17,18]. Stickleback at this latitude usually live for 2 years [19], and the fish collected were in their second spawning season at close to their maximum body size and lifespan. Fish were sacrificed by overdose in MS222 (Tricaine S, Sigma-Aldrich, MI, USA) in lake water, eviscerated, and cut along a line from the anal to third dorsal spine on both sides. Samples were fixed in 4 per cent (m/v) paraformaldehyde in phosphate-buffered saline (PBS) overnight, washed several times in PBS and stored in PBS with 1 per cent (m/v) sodium azide until processed for histology. A 4 mm thick steak was prepared close to 0.7 length and 8 μm transverse frozen sections of the entire cross section were cut and stained with modified Harris haematoxylin solution as described for charr. The total number of fast muscle fibres was counted from high-quality digital photographs (×100). Owing to variable shrinkage, it was not possible to quantify FD.

### (c) Statistical analysis

Statistics were performed using SigmaPlot v. 11.0 (Systat Software Inc, San Jose, CA, USA). For Arctic charr, between-population differences in FN_max_ and FD were analysed using one-way ANOVA with pairwise multiple comparisons by the Holm–Sidak method (overall significance level equal to 0.05). Differences in body size and FN_max_ between pooled populations of large- and small-bodied fish were compared using a *t*-test. The relationship between FD and body mass was fitted using linear regression. In each case, the data passed tests for normality and equal variance. For sticklebacks, differences in body size and FN_max_ between pooled populations of large- and small-body size failed tests for normality and were analysed with a non-parametric Mann–Whitney Rank Sum test.

## 3. Results

### (a) Arctic charr

Adults of all the dwarf populations studied shared paedomorphic features, including a blunt-head, sub-terminal mouth and parr markings (figure 1*b*,*c*). The vast majority (less than 85%) of fishes over 8.5 cm length were sexually mature with ripe gonads (figure 1*b*,*c*). The largest individual wild-caught fishes ranged from 9.6 cm length and 6.7 g (S population) to 15.5 cm length and 31.3 g (M population) (electronic supplementary material, table S1). Larger individuals of the A, K, S and J, but not the M, P and H populations were obtained following laboratory rearing (electronic supplementary material, table S1), although in all cases, body size was less than one-third of that found for large-bodied populations.

**Figure 2.**
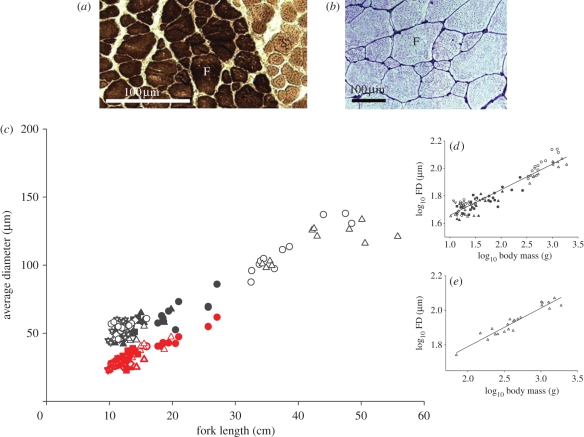
Axial muscle structure in wild-caught and laboratory-reared Arctic charr. (*a*) Cross section of a wild-caught dwarf charr from Álftavatn stained for myosin ATPase activity, illustrating darkly stained fast (F) and lightly stained slow fibres (S). (*b*) Cross section of a laboratory-reared dwarf charr from Álftavatn stained with haematoxylin showing the mosaic of fibre diameters (FDs) in fast muscle (F). (*c*) The relationship between body size and average FD for Arctic charr. Large-bodied populations: Lake Thingvallavatn large benthic morph (unfilled circles) and piscivorous morph (unfilled upright triangles) (data from Johnston *et al*. [6]). Dwarf populations in sympatry: Lake Thingvallavatn small benthic morph (data from Johnston *et al*. [6]) (filled circles), Álftavatn (filled upright triangles) and Silungapollur (filled squares). Dwarf populations in allopatry: Kaldárbotnar (unfilled circles), Miðhúsaskógur (unfilled upright triangles), Straumsvík (unfilled diamonds), Hrauná (unfilled stars) and Sílatjörn (unfilled inverted triangles). The equivalent red symbols show the estimated average FD calculated assuming FN_max_ was unchanged from the ancestral condition (see text for details). (*d*) Insert shows the log_10_ average fibre number plotted against log_10_ body mass for large-bodied and dwarf charr. Symbols are as for (*c*). (*e*) The scaling relationship for FD for the Thingvallavatn piscivorous morph including individuals at different stages of muscle fibre recruitment.

The arrangement of fast and slow myotomal muscle fibres in dwarf charr is illustrated in figure 2*a,b*. Slow muscle fibres (S) were lightly stained with myosin ATPase and restricted to a lateral strip on the surface of the myotome (figure 2*a*). Fast fibres (F) comprised the bulk of the myotome and had a wide range of FDs (figure 2*b*), reflecting continuous fibre recruitment during growth. The smallest diameter fast fibres were most resistant to alkaline pre-incubation, indicating expression of a developmental-stage-specific isoform of myosin in newly formed fibres (not shown).

FD and FN_max_ were analysed in the fast muscle of the largest wild-caught and laboratory-reared fishes from each population. Equivalent data were included for Arctic charr from Lake Thingvallavatn studied previously using identical methods [6]. Lake Thingvallavatn contains two benthic morphs, one of dwarf adult body size (8–22 cm, adult length; defined ‘small-benthic’, SB) that exploits sub-benthic habitats and the other of a relatively larger adult body size (20–50 cm; defined ‘large-benthic’, LB) that exploits epibenthic habitats, as well as two limentic morphs, one planktivorous (PL) (14–22 cm) and the other piscivorous (25–60 cm) (PI) [6,20] The absence of the smallest size class of diameters (less than 15 μm) was used to identify individuals that had stopped recruiting muscle fibres so that FN_max_ could be estimated. More than eight months of laboratory rearing of wild fish allowed maximal confidence in our FN_max_ estimates. The rationale is that environmental constraints (largely removed by laboratory rearing) may account for an absence of fibres less than 15 μm irrespective of genetic potential for further recruitment_._ Dwarfs from all the wild-caught populations except Álftavatn contained individuals that were at, or very close to, FN_max_. The smallest body length at which FN_max_ was established and the largest body length observed were 10.9 and 19.8 cm for A, 11.0 and 14.0 cm for J, 17.1 and 27.1 cm for SB, 11.1 and 14.1 cm for S, 9.8 and 13.0 cm for H, 11.3 and 17.1 cm for K, 10.5 and 15.5 cm for M and 11.3 and 13.5 cm for J.

The relationship between body length and the average diameters of fast muscle fibres is shown in figure 2*c*. For all populations, the relationship between log_10_ body mass and log_10_ FD fitted the equation:
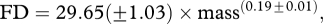
(mean ± s.e., adj Rsqr = 0.89, *F*_1,103_ = 798.3; *p* < 0.001; figure 2*d*). The scaling of FD for all stages of the PI morph is shown in figure 2*e* (FD = 21.98 (±1.10)×mass^(0.22^^±^^0.02)^, mean ± s.e., adj. Rsqr. = 0.91, *F*_1,20_ = 206.0; *p* < 0.001).

**Figure 3.**
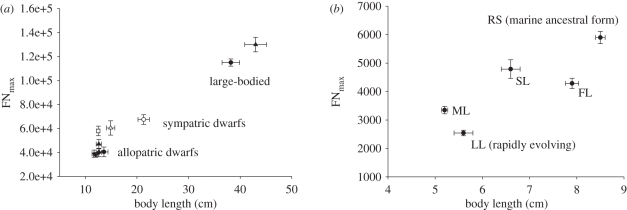
(*a*) The relationship between FN_max_ and body length for large-bodied and dwarf arctic charr populations. Symbols are as described in the legend to figure 2*c*. (*b*) The relationship between FN_max_ and body length for large-bodied and small-bodied threespine stickleback populations. RS: Rabbit Slough (*n* = 7), SL: Seymour Lake (*n* = 6), FL: Frog Lake (*n* = 8), ML: Mud Lake (*n* = 5), LL: Loberg Lake (*n* = 9), Alaska. Bidirectional error bars represent mean ± s.e. of the mean.

The mean value of FN_max_ for individuals across all dwarf populations was 50 900 ± 1000 (mean ± s.e., *n* = 73 fish), equivalent to an 84.5 per cent reduction in fibre number relative to the aquaculture strain (324 000 ± 30 000, mean ± s.e., *n* = 6 fish) and 60.5 per cent compared with the PI Thingvallavatn morph (figure 3*a*). Next, we tested the hypothesis that the degree of fibre loss would be less for sympatric than allopatric dwarfs. The average value for FN_max_ for sympatric dwarfs (59 000 ± 3300, *n* = 25; A, P and SB) was 43 per cent greater than for allopatric dwarfs (41 000 ± 1500, *n* = 41; K, M, S, H and J) (*p* < 0.0001). There were no significant differences in FN_max_ between the allopatric dwarf populations (K, M, S, H, and J; figure 3*a*).

### (b) Threespine stickleback

The average length and FN_max_ were 5.5 ± 0.1 cm and 2900 ± 130, respectively, for two small-bodied populations (ML, LL; mean ± s.e., *n* = 15). Individuals from three large-bodied populations (RS, SL, FL; 7.7 ± 0.2 cm body length) had an average FN_max_ of 5000 ± 200 (mean ± s.e., *n* = 21). Thus, there was a 42 per cent reduction in FN_max_ in the small- relative to large-bodied populations (mean ± s.e., *n* = 15; *p* < 0.01; figure 3*b*). Variation in FN_max_ was significant across all populations (*p* < 0.001). The RS population had a higher FN_max_ and greater body size than all other populations (*p* = 0.002 for RS versus SL and *p* < 0.001 for other comparisons), as did SL and FL versus the ML and LL populations (*p* < 0.01).

## 4. Discussion

Across a range of Arctic charr populations, FD scaled with a mass exponent of 0.19, irrespective of maximum observed body size (10–105 g; figure 2*d*). The fish studied inhabit broadly similar thermal environments [15], enabling direct comparisons of oxygen requirements between the different populations. According to the classical studies of A.V. Hill, the maximum radius (*Ro*) that oxygen can penetrate in a long circular cylinder of muscle for a given oxygen concentration at its surface (*Yo*) can be represented by the simplified equation:
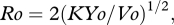
where *K* is Krogh's diffusion constant and *Vo* is the mass-specific oxygen consumption of the muscle [21]. Mass-specific resting metabolic rate was shown to have a mass exponent of 0.22 in six salmonid species [22]. Thus, the increase in FD with body mass in Arctic charr populations showed a close inverse relationship with the scaling of mass-specific resting metabolism. A similar scaling relationship of FD with a mass exponent of 0.22 was observed for the large-bodied PI morph when all fish (70–1893 g body mass, *n* = 22) were considered, including those still recruiting fibres (figure 2*e*). Thus, FD is the same for contemporary dwarf and large-bodied Arctic charr populations when corrected for differences in body mass, which is predicted by the OFS hypothesis. In species with indeterminate growth, FD is determined by the production of new muscle fibres, a process that continues into adult stages. The optimization of fibre size in dwarf Arctic charr was associated with an average reduction in FN_max_ of 60.5 per cent across all dwarf populations relative to the large-bodied PI morph. In turn, FN_max_ was 2.5-fold lower in the PI morph than the aquaculture strain of charr, which had been under selection for large size at age for six to seven generations. Dramatic intraspecific changes in fibre number were also observed in the threespine stickleback (figure 3*b*), which has markedly different life-history characteristics, consistent with selection for fibre size being a universal feature of teleost body size evolution.

To estimate the energy savings associated with fibre size optimization, we can calculate what FD would have been for dwarf charr lacking adaptive changes in FN_max_, taking the PI morph as a proxy ancestral state (red symbols in figure 2*c*). For the dwarf Arctic charr in allopatry (10–48 g), the average contemporary FD (μm) was 52.8 ± 0.8 compared with just 39.2 ± 0.9 in the absence of a reduction in FN_max_ (mean ± s.e., *n* = 45). Thus, the reduced muscle fibre production in dwarf charr resulted in a 34.7 per cent lower surface to volume ratio relative to the non-adapted state, which would be expected to produce equivalent reductions in the energy costs of maintaining ionic homeostasis [9]. It has been estimated that maintaining ionic homeostasis contributes 20–40% to resting metabolic rate in teleosts [23]. This suggests that a major selection pressure shaping FN_max_ evolution is likely to be energetic resource optimization, allowing maximal investment in other phenotypic traits contributing to Darwinian fitness.

Sympatric populations have a greater possibility of historical or contemporary gene flow with large-bodied morphs and this introgression would be expected to oppose divergent selection [16,24]. Arctic charr exhibit polygamous mating patterns typical of salmonids, in which males either guard a female or attempt sneak-mating with guarded females [25]. In Thingvallavatn, the nocturnally active dwarf benthic charr were observed to enter the nests of the larger benthic morph [25]. Thus, sneaking mating behaviour is a plausible mechanism for limited gene flow between charr morphs. In support of this idea, FN_max_ in the three sympatric populations that co-reside with large-bodied charr was significantly higher than for dwarfs living in allopatry (figure 3*a*).

The repeated evolution of muscle fibre recruitment traits in teleost populations may occur by selection acting on standing allelic variation or new mutations at loci with large effects, as demonstrated for several morphological traits [26–29]. Considering the apparent rapidity of the evolution of muscle fibre size optimization, as well as its occurrence in distantly related taxa, a limited number of conserved genes may have a large effect on evolutionary patterns of myogenesis. One candidate pathway involves insulin-like growth factor (IGF)—mechanistic target of rapamycin (mTOR) signalling, which relays environmental signals to cells to regulate growth and body size in diverse animal lineages [30–32]. This pathway also controls muscle fibre production in vertebrates by stimulating myoblast proliferation and differentiation [33]. By manipulating inputs to this pathway in the muscle of replicated populations of dwarf and large-bodied charr morphs, we obtained evidence for adaptive modification in the expression of several genes, including *mTOR* [34]. Allelic variation and/or de novo mutations in IGF–mTOR pathway genes and/or their upstream regulators would thus appear to be promising candidates on which selection might act during body size evolution. Whatever the precise mechanism, parallel evolution of myogenesis is likely to result from universal scaling laws affecting muscle fibre dimensions and energy metabolism.
